# Differential improvements between men and women in repeated CrossFit open workouts

**DOI:** 10.1371/journal.pone.0283910

**Published:** 2023-11-28

**Authors:** Gerald T. Mangine, Nina Grundlingh, Yuri Feito

**Affiliations:** 1 Exercise Science and Sport Management, Kennesaw State University, Kennesaw, Georgia, United States of America; 2 Data Science and Analytics, Kennesaw State University, Kennesaw, Georgia, United States of America; 3 American College of Sports Medicine, Indianapolis, Indiana, United States of America; Liverpool John Moores University, UNITED KINGDOM

## Abstract

**Introduction:**

The CrossFit^®^ Open (CFO) acts a preliminary round that qualifies men and women for later stages of its annual Games competition. The CFO typically consists of 4–6 workouts that variably challenge an athlete’s weightlifting strength, gymnastic skill, and endurance capacity. Except for differences in prescribed intensity loads, workouts are designed the same for men and women to elicit a similar challenge. While all workouts within a single year are unique to each other, one has been repeated from a previous CFO each year between 2012 and 2021. Because previous CFO workouts are often integrated into training, improvements are expected when a workout is officially repeated. However, besides documented record performances, it is unclear whether most athletes are improving, if these improvements affect ranking, or if differences exist between men and women.

**Purpose:**

To examine sex-division differences and performance changes across repeated CFO workouts, as well as their effect on CFO and workout ranking.

**Methods:**

Eleven separate samples of 500 men and 500 women, who were representative of the same overall percent rank within each year involving one of the nine repeated CFO workouts (2011–2021) were drawn for this study. Each athlete’s age (18–54 years), rank (overall and within each workout), and reported workout scores were collected from the competition’s publicly-available leaderboard. Each sample had excluded any athlete who had not met minimum performance criteria (e.g., at least one completed round) for all prescribed (Rx) workouts within a given year (including those not analyzed). Since some workouts could be scored as repetitions completed or time-to-completion (TTC), and because programming was often scaled between men and women, all scores were converted to a repetition completion rate (repetitions divided by TTC [in minutes]).

**Results:**

Separate sex-division x time analyses of variance with repeated measures revealed significant (*p* < 0.05) interactions in all but one repeated workout comparison. Initially, men were faster in four workouts (~18.5%, range = 3.9–35.0%, *p* < 0.001), women in two (~7.1%, range = 5.2–9.0%, *p* < 0.001), and they tied in the remaining three workouts. When workouts were repeated in subsequent years, men were faster in three workouts (~5.4%, range = 0.9–7.8%, *p* < 0.05), while women were faster in two (~3.8%, range = 3.5–4.1%, *p* < 0.01). Though performance improved in seven of the nine workouts (~14.3%, *p* < 0.001) and percentile rank was controlled, athletes earned a lower rank (overall and within workout) on each repeated workout (*p* < 0.001).

**Conclusions:**

Performance (measured as repetition completion rate) has improved in most repeated CFO workouts, particularly for women. However, improvements seen among all athletes, along with increased participation, have made it more difficult for athletes to improve their overall rank. To rank higher, individual athletes must improve their pace to a greater degree than the average improvements seen across the competitive field.

## Introduction

The CrossFit^®^ Open (CFO) has acted as the initial online qualification round for the CrossFit^®^ Games competition since 2011 [1]. The 3–5-week CFO has typically consisted of 4–6 workouts that variably challenge an athlete’s weightlifting strength, gymnastic skill, and endurance capacity [2, 3]. The details of each workout are announced weekly, and competitors may rely on the fact that all workouts within a particular year will be unique to each other. However, except in 2011 and 2022, each CFO competition has included a workout drawn from a previous competition, with nine distinct workouts acting as repeats between 2012 and 2021. Adapted from [2], the composition and movement standards for these workouts are presented in Tables 1 and 2, respectively.

**Table 1:** Repeated CrossFit^®^ open workouts.

Duration	Details	Appearances (scoring)		
10-minute AMRAP	30 x Double-unders	11.1 (repetitions)	14.1 (repetitions)	
	15 x Power snatches (75lbs / 55lbs)			
7-minute AMRAP	3 x Thrusters (100lbs / 65lbs)	11.6 (repetitions)	12.5 (repetitions)	18.5 (repetitions)
	3 x Chest-to-bar pull-ups			
	*Add 3 repetitions after each completed round			
12-minute AMRAP	150 x Wall ball shots (20lbs / 14lbs to 10’ / 9’ target)	12.4 (repetitions)	13.3 (repetitions)	
	90 x Double-unders			
	30 x Muscle-ups			
3-minute rounds (indefinite)	*Complete two sets of*:	14.2 (repetitions)	15.2 (repetitions)	
	10 x Overhead squats (95lbs / 65lbs)			
	10 x Chest-to-bar pull-ups			
	*Add 3 minutes and 2 repetitions for each completed round			
TTC	*21-18-15-12-9-6-3 repetitions of*:	14.5 (TTC)	16.5 (TTC)	
	Thrusters (95lbs / 65lbs)			
	Bar-facing burpees			
4-minute rounds (20-minute time limit)	25 x Toes-to-bar	16.2 (TTC or repetitions)	19.2 (TTC or repetitions)	
	50 x Double-unders			
	Squat cleans (R1: 135lbs / 85lbs x 15; R2: 185lbs / 115lbs x 13; R3: 225lbs / 145lbs x 11; R4: 275lbs / 175lbs x 9; R5: 315lbs / 205lbs x 7)			
	*Add 4 minutes for each completed round			
13-minute AMRAP	55 x Deadlifts (225lbs / 155lbs)	16.4 (repetitions)	17.4 (repetitions)	
	55 x Wall ball shots (20lbs / 14lbs to 10’ / 9’)			
	55 x Calorie rowing			
	55 x Handstand push-ups			
20-minute time limit	*Alternate the following*:	17.1 (TTC or repetitions)	21.2 (TTC or repetitions)	
	Dumbbell snatches (50lbs / 35lbs) x 10-20-30-40-50 repetitions			
	15 x Burpee box jump-overs (24" / 20")			
9-minute time limit	*Complete 21-15-9 repetitions of*:	18.4 (TTC or repetitions)	20.3 (TTC or repetitions)	
	Deadlifts (225lbs / 155lbs)			
	Handstand push-ups			
	*Then complete 21-15-9 repetitions of*:			
	Deadlifts (315lbs / 205lbs)			
	Handstand walk (50’)			

Notes: AMRAP = ‘as many repetitions as possible’; TTC = time to completion

**Table 2:** Exercise movement standards.

Exercise	Movement standards
Bar-facing burpees	Athlete begins standing perpendicular to and facing the barbell, and then drops to the floor, ensuring that the chest and thighs touch the ground. The athlete then lifts themselves to jump over the barbell from both feet and land on both feet. The next repetition will begin on the opposite side facing the barbell.
Burpee box jump-overs	Athlete begins standing perpendicular and facing the box, and then drops to the floor, ensuring that the chest and thights touch the ground. The athlete then lifts themselves to jump onto the box and drop to the floor on its opposite side, or jump completely over (not around) the box. A two-foot jump and landing is required, and then next repetition beings on the opposite side facing the box.
Calorie rowing	The monitor on a Concept2^™^ rower must be set to zero at the beginning of each row; it may be reset by the athlete or judge. The athlete must remain seated throughout the entire row until the targeted calories or distance may be clearly read on the monitor.
Chest-to-bar pull-ups	Athletes begin hanging from a standard pull-up bar with arms extended and feet off the ground and then pull themselves vertically so that their chest touches the bar before returning to the start position. “Kipping” or “butterfly” techniques are acceptable so long as the arms return to full extension at the bottom of each repetition.
Deadlifts	Using a traditional stance (i.e., hands outside the knees), athletes pick up a loaded barbell from the floor until their hips and knees reach full extension with the head and shoulders behind the bar and arms straight throughout the movement.
Double-unders	Using a jump rope, the athlete must spin the rope forward so that it completely passes under the feet twice during a single jump.
Dumbbell snatches	Athlete lift the dumbbell from the ground using one motion to finish with it directly overhead with arms, hips, and knees fully extended. The athlete then lowers the dumbbell so that both of its heads touch the ground. Athletes must alternate arms after each repetition and the non-lifting hand and arm may not be in contact with the body during the repetition.
Handstand push-ups	All repetitions begin and end at the top of a handstand with the arms and hips extended, with their hands within the pre-marked square on the ground, and heels contacting the wall at or above the pre-marked foot line. Athletes lower themselves so their head makes contact with the ground before returning to the starting position; "kipping" is permitted.
Handstand walk	Using a walking lane marked in 5-ft segments, athletes kick up with both hands completely behind the segment marking line. They must walk forward on their hands, while supporting the rest of their body in the air, until their hands completely pass the next segment line.
Muscle-ups	Athletes begin hanging from a standard pull-up bar with arms extended and feet off the ground and then pull themselves vertically so that their arms are extended in a support position above the bar, with shoulders over or slightly in front of the bar. "Kipping" is permitted, but pull-overs, rolls to support, and glide kips are not, and no portion of the foot may rise above the lowest part of the bar during the kip.
Overhead squats	The athlete lifts the loaded barbell from the floor to overhead with the hips, knees, and arms fully extended, and the bar directly over the body’s midline. The athlete maintains the overhead barbell position as they lower their body into a full squat (i.e., crease of the hip clearly passes below the top of the knees) before returning to the start position. A full squat snatch is permitted on the first repetition.
Power snatches	The athlete lifts the loaded barbell from the ground using one motion to bring it overhead with the hips, knees, and arms in full extension and the barbell directly over the body’s midline. The athlete then lowers the barbell so that both sides’ plates touch the ground; bouncing is prohibited.
Squat cleans	The athlete lifts the loaded barbell from the ground, extending their ankles, knees, and hips. The athlete must recieve the barbell in the front rack position either while in a full squat (i.e., (i.e., crease of the hip clearly passes below the top of the knees) or higher (i.e., power clean) and then lowering themselves into the full squat (i.e., power clean to front squat). From the full squat, the athlete must stand with their hips and knees are fully extended, before returning the barbell to the floor.
Thrusters	A loaded barbell is picked up from the floor into the front rack position and the athlete descends to a full squat (i.e., crease of the hip clearly passes below the top of the knees), returns to the starting position, and then immediately progresses into an overhead press with knees, hips, and arms at full extension with the barbell overhead.
Toes-to-bar	Athletes begin hanging from a standard pull-up bar with arms extended and feet off the ground before bringing their heels behind the bar and then swinging both feet simultaneously forward and up to touch the bar.
Wall ball shots	A medicine ball is picked up from the floor into the front rack position and the athlete descends to a full squat (i.e., crease of the hip clearly passes below the top of the knees), returns to the starting position, and then immediately progresses into a shooting motion to throw the ball so that its center hits a target at or above the specified height.

After their introduction, CFO workouts become benchmarks (i.e., named workouts that are commonly known across training facilities) that may be integrated into training. Although the CrossFit^®^ strategy aims to constantly vary the training stimulus to promote simultaneous and generalized fitness improvements [4], benchmark workouts will appear more often throughout training. Thus, it is reasonable to expect that greater familiarity with these workouts would lead to improved scores whenever one is officially repeated in competition [5, 6]. As one becomes more familiar with the physical tasks appearing in sport, they learn to activate more muscle when given the same tasks and eliminate inefficient and unnecessary actions [7–9]. Greater experience affords more opportunities to develop relevant skills and strategies that affect one’s approach to a workout (i.e., pacing strategy) and its resultant physiological demand. This is relevant to CrossFit^®^ competition because workouts are commonly structured to produce a score that emphasizes maximal workout density. Athletes are scored by their time-to-complete (TTC) assigned work or they are tasked with completing ‘as many repetitions as possible’(AMRAP) of assigned exercises within a set time limit [4, 10]. Efficiently performing movements and transitioning between exercises is at least partially responsible for an individual’s ability to maximize their score, and this ability can only be developed with practice. However, aside from documented record performances from the sport’s top athletes in repeated CFO workouts [2, 3], it is unclear whether improvements have occurred throughout the remaining competition pool.

Another important consideration is the impact of increased participation, particularly within the women’s division, on performance and ranking. The initial CFO pool of 26,000 athletes consisted of 13,127 Rx competitors (i.e., non-scaled or age-group athletes who completed all CFO workouts as prescribed) and 4,506 people competed in the women’s division (34.3%), and that number grew to 42,799 in 2021 (36.7%) [11]. Though the same absolute rank becomes mathematically less important with more people vying for the same number of spots at the Games, the effect on percentage-rank remains unclear. It is also unclear whether such an effect, if present, has been equal across the men’s and women’s competitive divisions.

Unlike more traditional sports, the only observable difference between the men’s and women’s divisions are participation and programming [12]. With programming, workouts are often scaled, presumably to account for known physiological differences between biological men and women [2, 3]. However, scaling practices are not uniformly prescribed and their effect on performance has not been specifically explored. Among repeated CFO workouts, weightlifting intensity loads were the most often scaled programming feature with loads assigned to women ranging between 62.2% and 73.3% of those assigned to men, including wall ball shot target distance and box jump height [2]. While scaling resistance-based exercises may account for known strength and power differences [13], this rationale has not been extended to gymnastic-calisthenic exercise prescription (e.g., handstand push-ups/walking, burpees, pull-ups, and muscle-ups), though difficulty may be naturally equated for these. Gymnastic-calisthenic exercises primarily require the athlete to maneuver their own body mass about an object (e.g., pull-up bar, rings, box) [4, 10] and women tend to possess less body mass [13, 14]. In contrast, scaling has yet to be applied to jumping rope and rowing tasks, which seems to ignore known sex differences in physiological measures of cardiorespiratory fitness [13, 15]. It remains unknown whether these programming decisions have been successful in equating the challenge presented to competitors in the men’s and women’s divisions, nor if this has changed over time.

In non-CFO workouts, one study reported no differences between 13 men and 10 women for a 20-minute AMRAP, as well as a TTC version of the same workout, that scaled rowing calories, deadlift loads, and kettlebell swing loads but not burpees [16]. However, the small sample size affected the generalizability of those findings and experience (the only available proxy of athlete skill) was significantly different between sexes and could have affected how each approached the workouts. Conversely, normative scores were developed from very large random samples of CFO competitors (*n* = 7,046–89,792) for all workouts programmed between 2011 and 2022 [17], and performance differences between men and women were noted in 56 out of 60 total workouts. Although this implies ineffectual CFO scaling, it would be premature to definitively make this conclusion due to unequaled sample sizes and participant skill. Equating these would provide a better understanding about whether scaling practices have led to similar scores in the men’s and women’s divisions. In turn, this information would assist in developing generalized training recommendations and provide better context for studies aiming to determine physiological predictors of performance. Thus, the purpose of this investigation was to begin that process by examining performance differences between similarly-ranked competitors within the men’s and women’s divisions, and their changes over time, in repeated CFO. A secondary aim was to determine how changes in performance translated to ranking in each workout in each workout and the overall competition.

## Methods

### Study design

To determine differences between men’s and women’s performances, as well as their changes over time in repeated CFO workouts, performance data was collected for all athletes participating in CFO competitions from 2011 to 2021. All competition results were obtained from the JavaScript Object Notation (JSON) file located on the publicly-available, official competition leaderboard [11]. Since these data were pre-existing and publicly available, the Kennesaw State University’s Institutional Review Board classified this study as exempt (IRB# 16–215), and participants did not have to provide their informed consent. Python3 was used to convert the data into a CSV format and treated in Microsoft Excel (v. 365, Microsoft Corporation, Redmond, VA, USA). Treating the data involved removing all age-group athletes (e.g., teens and masters) and cases that did not meet study inclusion criteria. The retained data included each athlete’s age and final overall ranking (i.e., official CFO rank awarded within a given year), as well as their official rank and score for each repeated CFO workout that they completed. Subsequently, differences were examined between divisions and repeated efforts.

### Participants

Based on pilot data [18] and the expectation of a small effect (Effect of *f* = 0.10), an a priori analysis using G*Power (v. 3.1.9.7, Heinrich-Heine-Universität, Germany) for a repeated-measures design indicated at least 328 participants would provide sufficient power (α = 0.05, β = 0.95) to observe differences between divisions and performances in each repeated CFO workout. Since collecting data on the same 328 men and women from 2011 to 2021 would produce a very specific sample, one that is likely representative of a subset of CFO participants, this study identified cases based on percent rank and made comparisons between athletes of the same percent rank across competition years.

Initially, a stratified list of 500 percentile ranks were identified based on the approximate percentage of cases that would fall within each standard deviation (SD) bin: 0.0–0.5 SD (38.2%, *n* = 192), 0.5–1.0 SD (30.0%, *n* = 150), 1.0–1.5 SD (18.4%, *n* = 92), 1.5–2.0 SD (8.8%, *n* = 44), 2.0–2.5 SD (3.4%, *n* = 16), 2.5–3.0 SD (1.0%, n = 4), and 3.0–3.5 SD (0.2%, *n* = 2) [19]. Ranks were evenly divided within each bin and across each side of the mean (e.g., –2.75 SD, –2.5 SD, +2.5 SD, and +2.75 SD). These percent-ranks would be used to identify the specific cases within each CFO year that would be drawn from the pool of athletes who also met study criteria. Age, rank, and performance data were retained for all athletes, between the ages of 18 and 54 years (i.e., non-age group athletes), who completed all CFO workouts as prescribed (i.e., as Rx with no within-division scaling) within a specific competition year. Additionally, cases were excluded if they did not complete at least one round (in AMRAP-style workouts), the first exercise couplet (in repeated couplet workouts), or all repetitions assigned for the first exercise (for TTC or when multiple rounds were not expected) for every workout within a single competition year. These criteria were meant to limit the inclusion of workout “specialists” and those who did not intend on completing or could not perform the exercises for the Rx workout. To match specific cases with the identified percent ranks to be drawn from within each year’s athlete pool, retained athletes were ordered based on their final within-division overall competition rank and then assigned a within-year percent rank (i.e., percent rank among athletes meeting study criteria). The final within-year percent ranking was used to identify 500 athletes within each year who would be included in this study. Thus, the same array of percent-ranks was represented by the athletes retained each year. Table 3 provides a summary of the initial pool of competitors for each year, the number of cases meeting study criteria, and the age and final competition ranking characteristics of those retained for analysis.

**Table 3:** Case selection and sample characteristics.

Competition		Cases			Age (y)	Overall Rank
year	class	Initial	Met criteria	Included	mean ± SD (range)	mean ± SD (range)
2011	Women	4,506	3,046	500	30.2 ± 6.3 (18–46)	2,055 ± 1,234 (7–4,475)
	Men	8,621	7,046	500	29.7 ± 6.2 (18–47)	4,121 ± 2,454 (15–8,612)
2012	Women	14,217	8,621	500	30.9 ± 5.8 (18–44)	4,591 ± 2,843 (18–11,976)
	Men	25,027	18,873	500	30.7 ± 5.6 (18–44)	9,766 ± 5,799 (38–21,842)
2013	Women	32,643	14,144	500	31.7 ± 6.8 (18–54)	7,987 ± 5,320 (29–24,946)
	Men	52,169	36,808	500	31.5 ± 6.8 (18–54)	19,123 ± 11,424 (74–44,964)
2014	Women	52,076	36,863	500	30.9 ± 7.1 (18–54)	14,680 ± 9,402 (54–41,964)
	Men	80,284	63,828	500	31.6 ± 7.1 (19–54)	32,598 ± 19,038 (128–70,200)
2015	Women	108,764	7,802	500	29.3 ± 5.9 (17–53)	4,995 ± 3,849 (16–22,305)
	Men	153,272	45,615	500	30.4 ± 6.7 (18–50)	24,583 ± 15,474 (92–65,527)
2016	Women	130,154	16,372	500	30.3 ± 6.3 (18–51)	9,856 ± 7,014 (33–35,206)
	Men	178,510	53,920	500	31.1 ± 6.9 (18–52)	28,340 ± 17,430 (108–76,861)
2017	Women	159,563	36,721	500	31.9 ± 6.8 (18–52)	20,271 ± 13,222 (74–62,666)
	Men	214,519	84,669	500	32.2 ± 7.0 (18–52)	49,020 ± 32,240 (170–136,725)
2018	Women	171,976	31,007	500	31.7 ± 7.0 (18–51)	17,955 ± 12,368 (63–62,929)
	Men	227,562	78,268	500	32.5 ± 7.0 (18–53)	44,752 ± 29,760 (157–137,436)
2019	Women	146,363	39,895	500	32.5 ± 7.2 (18–54)	22,558 ± 15,109 (80–72,134)
	Men	195,562	87,197	500	33.3 ± 7.4 (18–54)	50,950 ± 33,569 (174–140,343)
2020	Women	94,157	20,965	500	33.3 ± 7.8 (18–53)	12,372 ± 8,793 (42–45,870)
	Men	133,874	51,394	500	33.7 ± 7.1 (18–53)	29,343 ± 19,410 (103–90,568)
2021	Women	108,641	42,799	500	33.6 ± 7.5 (18–54)	22,426 ± 13,470 (91–53,507)
	Men	137,464	73,750	500	33.1 ± 7.1 (18–54)	21,708 ± 12,537 (86–43,846)

### Workout descriptions

The specific programming details for each workout included in this study are provided in Tables 1 and 2. During CFO competition, workouts were individually released on each week of the competition, and athletes have predominantly been given four days to submit their best score to competition officials (e.g., Thursday evening to Monday evening). To be recognized as valid, any submitted attempt must have either been completed at a CrossFit^®^ affiliate in front of a judge who passed the online Judge’s Course, or filmed using standardized criteria [3]. Once a submission period ends, competition officials review and certify attempts, and award each a final rank and point value. Because all data were collected from the official competition leaderboard [11], this study assumed that all workout criteria and movement standards had been met and verified by competition officials. The data retained for analysis included the athlete’s official rank for each workout and score, recorded as TTC or repetitions. For uniformity and to enable fair comparisons when a workout’s official score could be stated as TTC or repetitions completed (i.e., when a TTC workout had a time limit to complete all work), all workout scores were converted into a repetition completion rate (i.e., repetitions completed divided by TTC or workout duration; repetitions x minute^-1^), as previously described [20]. Regardless of the specific workout’s scoring format, a greater repetition completion rate would always indicate a better competition score. This metric was also used to calculate each athlete’s percent-rank within each workout.

### Statistical analysis

Initially, the assumption of normal distribution was verified by the Shapiro-Wilk test. Subsequently, separate two-tailed, two-way (Time x Sex Division) analyses of variance with repeated measures were performed for each repeated CFO workout comparison. Dependent variables included age and original overall rank (within each year) and original workout rank, calculated percent-rank, and repetition completion rate. Except for the instance when a CFO workout was repeated twice (i.e., 11.6 vs. 12.5 vs. 18.5), sphericity was assumed for all repeated workout comparisons. For the exception, the Greenhouse-Geisser correction was applied because sphericity was violated on each comparison. All significant main effects and interactions were further assessed by pairwise comparisons using the Bonferroni adjustment. Effect sizes (η^2^_P_: Partial eta squared) were also used to quantify the magnitude of any observed differences [21]. Interpretations of effect size were evaluated at the following levels: small effect (0.01–0.058), medium effect (0.059–0.137) and large effect (> 0.138). All statistical analyses were performed using SPSS (v. 28.0, SPSS Inc., Chicago, IL). Significance was accepted at an alpha level of *p* ≤ 0.05, and all data are reported as mean ± SD.

## Results

### Ranking and athlete age

No differences were seen in the workout percent-rank calculated for each repeated workout within each comparison, except for a main effect for sex division when comparing 12.4 and 13.3 (F = 17, *p* < 0.001, η^2^_p_ = 0.02). Workout percent-rank was greater in men (29.8 ± 27.1%) than women (27.1 ± 23.5%) during the workout’s original and repeated appearances. In contrast, significant time x sex division interactions (F = 14–1089, *p* < 0.001, η^2^_p_ = 0.01–0.52) were noted with original overall rank for each workout comparison. In each comparison, men ranked lower (i.e., farther from first place) within their division than women and ranking further declined whenever a CFO workout was repeated. Likewise, the original rank assigned for performance in each workout followed a similar pattern in each comparison.

Significant time x sex division interactions were only found in the comparisons between 11.1 and 14.1 (F = 4, *p* < 0.05, η^2^_p_ < 0.01) and between 11.6, 12.5, and 18.5 (F = 3, *p* < 0.05, η^2^_p_ < 0.01). Average age in men increased from 2011 to 2012 (+1.1 years, *p* < 0.05), from 2011 to 2014 (+ 2.0 years, *p* < 0.001), and from 2012 to 2018 (+1.8 years, *p* < 0.001), but only from 2011 to 2018 in women (+1.5 years, *p* < 0.001). Main effects for time, where age increased in subsequent years, were noted in all remaining comparisons except for between 14.5 and 16.5 (*p* = 0.053). Main effects for sex division were also noted with the comparisons between 14.2 and 15.2 (F = 9, *p* < 0.01, η^2^_p_ = < 0.01), 14.5 and 16.5 (F = 5, *p* < 0.05, η^2^_p_ < 0.01), and 16.2 and 19.2 (F = 6, *p* < 0.05, η^2^_p_ < 0.01), where men were older than women in each case. Comparisons between sex divisions across repeated CFO workouts for age, overall rank, workout rank, and workout percentile rank are presented in Table 4.

**Table 4:** Comparisons between age, overall rank, workout rank, and workout percent rank in repeated CFO workouts.

		Overall					Workout					
		Age			Rank		Rank			Percent Rank		
		Women	Men		Women	Men	Women	Men		Women	Men	
11.1 vs. 14.1	Year 1	30.2 ± 6.3	29.7 ± 6.2		2,055 ± 1,234	4,121 ± 2,454*	2,579 ± 1,782	5,550 ± 3,694*		49.6 ± 28.9	49.6 ± 28.9	
	Year 2	30.9 ± 7.1	31.6 ± 7.1#		14,680 ± 9,402#	32,598 ± 19,038*#	20,343 ± 15,373#	45,083 ± 29,251*#		49.6 ± 28.9	49.6 ± 28.9	
11.6 vs. 12.5 vs. 18.5	Year 1	30.2 ± 6.3	29.7 ± 6.2		2,055 ± 1,234	4,121 ± 2,454*	2,069 ± 1,259	4,076 ± 2,557*		49.3 ± 28.9	49.2 ± 28.9	
	Year 2	30.9 ± 5.8	30.7 ± 5.6#		4,591 ± 2,843#	9,766 ± 5,799*#	4,683 ± 2,931#	9,707 ± 5,895*#		49.3 ± 28.9	49.3 ± 29.0	
	Year 3	31.7 ± 7.0†	32.5 ± 7.0#		17,955 ± 12,368#	44,752 ± 29,760*#	19,959 ± 14,193#	47,381 ± 32,522*#		49.2 ± 29.1	49.2 ± 29.0	
12.4 vs. 13.3	Year 1	30.9 ± 5.8	30.7 ± 5.6		4,591 ± 2,843	9,766 ± 5,799*	4,311 ± 3,572	10,154 ± 6,084*		42.3 ± 25.4	47.7 ± 28.9	*
	Year 2	31.7 ± 6.8	31.5 ± 6.8		7,987 ± 5,320#	19,123 ± 11,424*#	9,943 ± 6,922#	22,347 ± 13,810*#		40.7 ± 26.0	48.2 ± 28.8	
		#										
14.2 vs. 15.2	Year 1	30.9 ± 7.1	31.6 ± 7.1	*	14,680 ± 9,402	32,598 ± 19,038*	15,582 ± 9,582	40,097 ± 24,873	*	49.2 ± 29.1	49.5 ± 29.0	
	Year 2	29.3 ± 5.9	30.4 ± 6.7		4,995 ± 3,849#	24,583 ± 15,474*#	6,310 ± 5,449	30,974 ± 21,526		49.3 ± 29.1	49.4 ± 29.1	
		#					#					
14.5 vs. 16.5	Year 1	30.9 ± 7.1	31.6 ± 7.1	*	14,680 ± 9,402	32,598 ± 19,038*	15,952 ± 10,846	32,977 ± 19,534	*	49.9 ± 28.9	49.9 ± 28.9	
	Year 2	30.3 ± 6.3	31.1 ± 6.9		9,856 ± 7,014#	28,340 ± 17,430*#	13,159 ± 10,463	31,034 ± 20,961		49.9 ± 28.9	49.9 ± 28.9	
							#					
16.2 vs. 19.2	Year 1	30.3 ± 6.3	31.1 ± 6.9	*	9,856 ± 7,014	28,340 ± 17,430*	11,820 ± 8,301	33,667 ± 21,619*		48.5 ± 29.1	48.2 ± 29.3	
	Year 2	32.5 ± 7.2	33.3 ± 7.4		22,558 ± 15,109#	50,950 ± 33,569*#	25,424 ± 16,165#	55,081 ± 33,401*#		49.0 ± 28.8	48.7 ± 28.9	
		#										
16.4 vs. 17.4	Year 1	30.3 ± 6.3	31.1 ± 6.9		9,856 ± 7,014	28,340 ± 17,430*	12,721 ± 11,796	33,985 ± 24,139*		49.1 ± 29.1	49.1 ± 29.1	
	Year 2	31.9 ± 6.8	32.2 ± 7.0		20,271 ± 13,222#	49,020 ± 32,240*#	24,992 ± 18,278#	53,371 ± 34,233*#		48.6 ± 29.6	49.0 ± 29.3	
		#										
17.1 vs. 21.2	Year 1	31.9 ± 6.8	32.2 ± 7.0		20,271 ± 13,222	49,020 ± 32,240*	29,551 ± 21,148	62,539 ± 41,623*		49.8 ± 29.0	49.8 ± 29.0	
	Year 2	33.6 ± 7.5	33.1 ± 7.1		22,426 ± 13,470#	21,708 ± 12,537*#	26,306 ± 16,692#	25,240 ± 16,729*#		49.8 ± 29.0	49.9 ± 28.9	
		#										
18.4 vs. 20.3	Year 1	31.7 ± 7.0	32.5 ± 7.0		17,955 ± 12,368	44,752 ± 29,760*	17,499 ± 10,649	45,516 ± 28,014*		49.1 ± 28.9	49.2 ± 29.1	
	Year 2	33.3 ± 7.8	33.7 ± 7.1		12,372 ± 8,793#	29,343 ± 19,410*#	12,450 ± 7,282#	30,647 ± 18,397*#		49.1 ± 29.1	49.2 ± 29.0	
		#										

Note:

* = Significant (*p* < 0.05) difference between men and women;

^#^ = Significant (*p* < 0.05) difference from previous year(s); † = Significant (*p* < 0.05) difference between 11.6 and 18.5

### Workout completion rate

**Fig 1:**
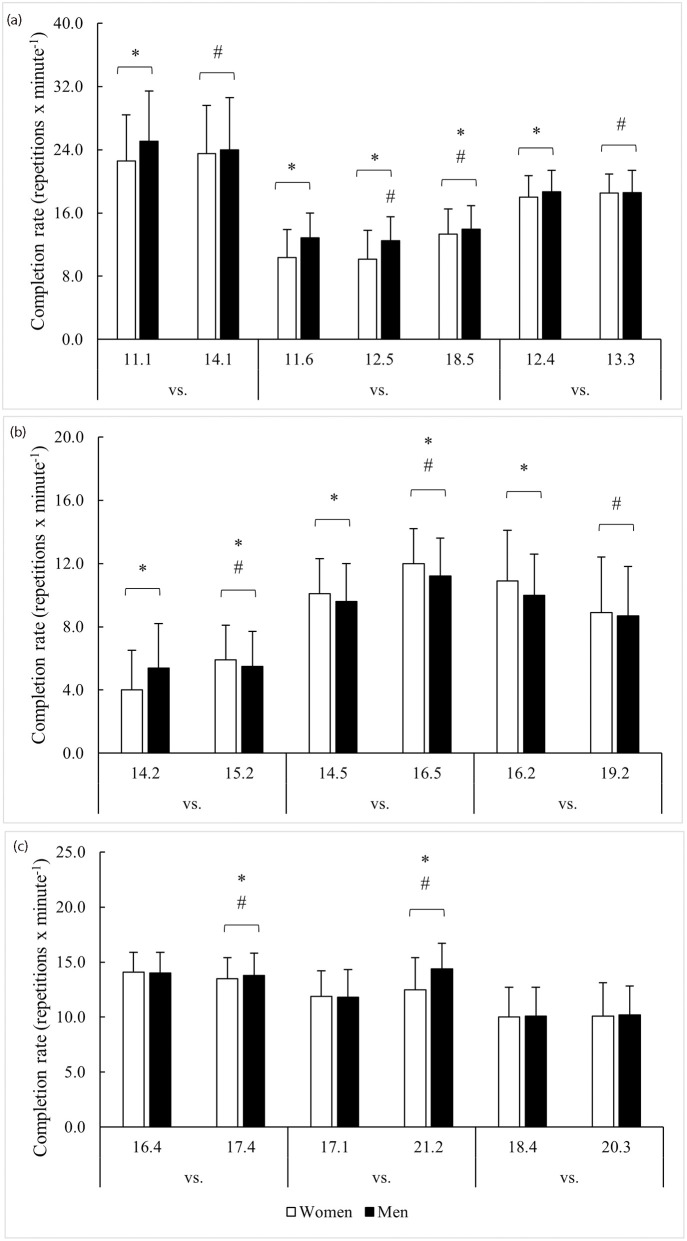
Repeated workout comparisons for repetition completion rate. Note: * = Significant (*p* < 0.05) difference between men and women; # = Significant (*p* < 0.05) difference from previous year(s).

Significant time x sex division interactions (F = 14–357, *p* < 0.001, η^2^_p_ = 0.01–0.26) were observed for all workout completion rate comparisons except for between 18.4 and 20.3, where no differences were found between sex divisions or repeated performance. Comparisons between sex divisions and across repeated CFO workouts for repetition completion rate are illustrated in Fig 1.

### 11.1 vs. 14.1

Though men completed 11.1 at a faster rate than women (+2.5 repetitions x minute^-1^, *p* < 0.001), no differences were seen in 14.1. In 2014, women improved repetition completion rate (+4.0%, *p* < 0.001), while men declined (–4.4%, *p* < 0.001).

### 11.6 vs. 12.5 vs. 18.5

Men completed the workout faster than women in each year (0.6–2.5 repetitions x minute^-1^, *p* < 0.001). However, compared to 2011, their performance declined in 2012 (–3.0%, *p* < 0.01) before improving by 8.4% in 2018 (*p* < 0.001). Women remained steady from 2011 to 2012 before improving in 2018 (+28.4%, *p* < 0.001).

### 12.4 vs. 13.3

Men completed 12.4 at a faster rate than women (0.7 repetitions x minute^-1^, *p* < 0.001) but not 13.3 (*p* = 0.703). In 2013, women improved their repetition completion rate by 0.5 repetitions x minute^-1^ (*p* < 0.001) and men slowed by 0.1 repetitions x minute^-1^ (*p* < 0.001).

### 14.2 vs. 15.2

Men completed 14.2 at a faster rate than women (1.4 repetitions x minute^-1^, *p* < 0.001) but slowed for 15.2 by 0.2 repetitions x minute^-1^ (*p* < 0.05). Women improved their repetition completion rate for 15.2 by 1.9 repetitions x minute^-1^ (*p* < 0.001) and exceeded the pace in men by 0.4 repetitions x minute^-1^ (*p* < 0.01).

### 14.5 vs. 16.5

Compared to men, a faster repetition completion rate was seen in women for both 14.5 (0.5 repetitions x minute^-1^, *p* < 0.001) and 16.5 (0.8 repetitions x minute^-1^, *p* < 0.001), with both men (1.6 repetitions x minute^-1^, *p* < 0.001) and women (1.9 repetitions x minute^-1^, *p* < 0.001) improving their speed from 2014 to 2016.

### 16.2 vs. 19.2

A faster repetition completion rate was seen in women for 16.2 (0.9 repetitions x minute^-1^, *p* < 0.001) but not 19.2 (*p* = 0.360). Though men performed 19.2 at a slower pace (–1.2 repetitions x minute^-1^, *p* < 0.001), women experienced a greater decline (–2.0 repetitions x minute^-1^, *p* < 0.001).

### 16.4 vs. 17.4

No differences in repetition completion rate were noted between men and women for 16.4 (*p* = 0.680). In 2017, men completed 17.4 at a faster rate than women (0.2 repetitions x minute^-1^, *p* < 0.05), but this was due to men slowing down less (–0.3 repetitions x minute^-1^, *p* < 0.001) than women (–0.6 repetitions x minute^-1^, *p* < 0.001).

### 17.1 vs. 21.2

No differences in repetition completion rate were noted between men and women for 17.1 (*p* = 0.441). In 2021, men completed 21.2 at a faster rate than women (2.0 repetitions x minute^-1^, *p* < 0.05), and this was due to men improving their pace by 2.6 repetitions x minute^-1^ (*p* < 0.001) compared to the 0.5 repetitions x minute^-1^ improvement (*p* < 0.001) seen in women.

## Discussion

This study examined performance differences between competitors within the men’s and women’s divisions in repeated CFO workouts and their effect on competition ranking. Out of nine separate repeated CFO workouts, performance improved in five workouts, diminished in two, and remained the same in one (i.e., the 9-minute AMRAP programmed for 18.4 and 20.3). Though their workout pace generally improved over time, the absolute rank awarded to competitors on the Leaderboard [11] declined. An outcome most likely due to increased participation over time because neither the competitors’ percent-rank overall nor within repeated workouts changed. Thus, to achieve a comparable or better percent-rank in a repeated workout, athletes would have had to improve performance beyond what was typically seen across all competitors. Additionally, initial, and subsequent performances were not equal among men and women. Men were initially faster in four of the workouts, while women outpaced them in two, and tied in the remaining three. Men were still faster in more workouts when they were repeated (3 workouts versus 2 workouts), but the specific workouts were slightly different. Men and women tied on the second iteration of two workouts where men were initially faster, while men were slower than women in a third workout where they had been initially faster. Women also tied men on the second iteration of a workout where they were initially faster. Aside from a preliminary conference presentation [18], this is the first study to investigate performance changes in official CrossFit^®^ competition workouts.

Improved performance was observed in six of the nine repeated CFO workouts. A learning effect would seem to be the most likely explanation for these improvements. Except when they are repeated, CFO workouts are novel and uniquely vary in exercise complexity, relative intensity, workload requirements, and structural design (i.e., AMRAP, TTC) [2, 3]. Though experience may facilitate the process [22–25], competitors may not be able to reconcile their own physiological and skill-related abilities with specific workout requirements on their first attempt. Moreover, any extra attempts would contribute to accumulated fatigue and damage that is not likely to have abated within the typical 4-day allotment [3, 20, 26], and this could negatively impact later efforts. Athletes may simply not find their ideal pacing strategy for each workout within a single CFO competition. However, after their introduction, CFO workouts may be integrated into training and/or frequently discussed (in-person or online) amongst athletes. With an average of 2.4 years separating repeated CFO workouts [5, 6], athletes have ample opportunities to refine their pacing strategy. Albeit they would not know which and when specific CFO workouts might be repeated, leaving an ever-growing list of potential workouts and skills in need of practice.

Having time to perfect pacing strategy may have been less relevant to performance in the three workouts that did not improve (i.e., 16.2–19.2, 16.4–17.4, and 18.4–20.3). Compared to all other workouts, these three required more strength and capacity to lift heavier loads or perform complex gymnastic movements for multiple repetitions. For instance, deadlift loads ranged from 225–315 lbs. (102.1–142.9 kg) for men and 155–205 lbs. (70.3–93.0 kg) for women, and power clean loads ranged from 135–315 lbs. (61.2–142.9 kg) for men and 85–205 lbs. (38.6–93.0 kg) for women. Further, these heavier loads were always paired with one or more gymnastics-calisthenic movements prescribed for multiple repetitions (e.g., 25 toes-to-bar, 50 double-unders, 45–55 handstand push-ups, and 150 feet [45.7 m] of handstand walking). In contrast, the highest load required in all other workouts was for thrusters (men: 100 lbs. [45.4 kg]; women: 65 lbs. [29.5 kg]), and these were typically paired with low-complexity calisthenics (e.g., burpees, burpee box jumps, and double-unders) prescribed at noticeably less volume. The only instance where gymnastics prescription was comparable involved 30 muscle ups (i.e., 12.4–13.3), and the average athlete did not even complete a full round of this workout. While modifying pacing would seem to have a more immediate effect on these latter workouts, improving upon the former would have required more time to develop strength and strength endurance, as well as acquire or improve upon relevant gymnastic skills [27–29]. On average, athletes had less time to work on relevant skills for these three repeated workouts (2.0 years) compared to the other workouts (2.6 years).

An alternate explanation for the observed performance changes involves the relative growth of the sport. Compared to the first CFO in 2011, the number of athletes presently meeting this study’s criteria increased 156–1,305% and 168–1,138% for women and men, respectively. Although the competitive aspect about CrossFit^®^ has been identified as a highly influential factor for participation and retention [30], little is known about the athletic histories of pre-existing and newly-introduced CFO participants. In a 2016 epidemiological survey of Brazilian CrossFit^®^ athletes [31], 70.5% of respondents reported being physically active on > 3 days per week doing a variety of activities (i.e., weight training, running, soccer, and martial arts) for several years. Skills learned and developed across various sports and levels of competition are known to have value for an athlete’s primary sport [8, 9], and such skills brought in by newcomers could at least partially explained the improved performances seen in this study. An intentional examination into the development of relevant skills, and whether they were obtained prior to or during CrossFit^®^ training, would provide greater insight into the factors responsible for the growth of this sport.

The factors responsible for the growth in CFO participation may also be relevant to the differences seen between men and women. In 2011, women accounted for 34.3% of all competitors (or 30.2% of competitors meeting this study’s criteria). Out of all the instances where men outpaced women, half (*n* = 4) were seen during the repeated workouts of the competition’s first two years. Since 2011, the percentage of competitors who were women increased by 0.8% per year; a 0.3% increase per year out of competitors who met this study’s criteria. Concomitantly, an equal number of instances (initial and repeated) where men (*n* = 4) or women (*n* = 4) performed better than the other were seen during this time. No differences were observed in all other instances (*n* = 7). Moreover, out of the four workouts where men initially performed better, women either eliminated or later outpaced men. Thus, participation in the women’s division is clearly increasing, and women appear to be experiencing greater improvements (~8.3% across all workouts) compared to men (~2.8% across all workouts). The driving force(s) behind these improvements is/are not well understood. It is possible that this observation is a simple mathematical function where lower values do not require dramatic additions in absolute numbers to experience larger percent increases. Alternative explanations include the potential lure of CrossFit^®^ and competition for women who possess athletic backgrounds [32], social and health (physical and mental) benefits [32, 33], or even a growing realization that this sport empowers women unlike more traditional sports [34, 35].

The presence and adequacy of program scaling are caveats to the observed sex-division differences with pacing. CrossFit^®^ modifies workout characteristics to presumably acknowledge known physiological differences between biological men and women and present a similar challenge [2, 3, 12]. If applied correctly, performance differences should not exist. However, there were differences in both performance and the application of scaling across exercise types. Resistance training loads, medicine ball weight and target distance, and box jump height were all scaled, but not gymnastic and calisthenic movements (e.g., chest-to-bar pull-ups, handstand push-ups, handstand walking, burpees, rope jumping), continuous exercise patterns (referred to as monostructural, e.g., rowing), or programming durations. Although scaled loads might account for strength differences [13], their arbitrary prescription assumes a specific strength difference that cannot be known across thousands of competitors. Likewise, known differences in body mass [14] might support why gymnastics and calisthenics are not scaled but fails to account for the known upper-body strength differences [13] that are relevant to several of these exercises. Finally, the lack of scaling for continuous exercises or workout durations ignore the typical differences seen with aerobic and anaerobic capacity [13, 15]. Thus, it can be concluded that scaling was not adequately applied, at least across the repeated CFO workouts examined in this study. Others are encouraged to build on these findings by specifically investigating the adequacy of scaled programming assigned to the men’s and women’s CFO divisions.

The present study’s findings are not without limitations. Competition rule and structure changes (e.g., specific days allotted to submit scores, number of workouts, submission card details), as well as variations in when each workout iteration appeared (e.g., the repeated workout 18.4–20.3 appeared on different weeks in 2018 and 2020), and the number of attempts individual athletes made before submitting their best score, have all affected the exact context in which each workout was performed. Nevertheless, these were unavoidable variations that are consistent with the training strategy [4] but should still be kept in mind when interpreting these results. Another limitation involved purposeful, stratified selection of cases based on percent rank to make fair comparisons between sex-divisions and repeated performances. The non-random stratification was done to best approximate a normally-distributed population [19] and since repeated performances were not followed in a random sample of the same exact athletes, it is possible that individual differences and variances in age and relevant physical, physiological (including biological men and women self-selecting to compete in the alternate’s sex division), and psychological traits influenced the analyzed scores [22–25, 36–40]. However, given the growth of CFO participation, it would not have been possible to follow a sufficient sample of the same athletes over a decade of competitions. Even if it were, that sample might be more accurately representative of a specific subset of more experienced CFO athletes, rather than a heterogenous representation of the overall CFO population. Conversely, the opposite rationale underpinned this study’s exclusion criteria. Cases were excluded if the athlete did not earn a score beyond a minimum threshold assigned to each CFO workout of a specific year. These measures were meant to ensure that our findings were representative of a homogenous sample of healthy (i.e., those that did not miss a workout due to injury), well-rounded (i.e., non-workout specialists or those attempting to boost their rank by only completing a small number of repetitions in a specific workout) CrossFit^®^ athletes. But in doing so, it is possible that representative, low-ranking cases were eliminated, and this could have slightly skewed our results. Finally, the validity of the extracted scores is ultimately reliant on determinations made by CFO competition officials [3]. As these cannot be verified, it is possible that the analyzed scores included errors in reporting, individual variation in meeting exercise movement standards, and outright cheating. Still, the conservative approach in estimating expected sample requirements for sufficient statistical power, and then exceeding the minimum sample size should have assuaged the impact of these limitations on our results.

The findings of this study suggest that performance (measured as repetition completion rate) in most repeated CFO workouts, particularly women, has improved since the competition’s inception. These data serve as initial documentation that, though often scaled, athletes within the men’s and women’s divisions scored differently in ~63.2% of workouts. These differences warrant a more in-depth look across a broader range of workouts, and more specifically, how they might affect acute and long-term physiological responses. Doing so, might help guide more effective, sex-division-equated scaling practices. From a competitive standpoint, the general improvements seen among athletes, along with increased participation, have made it more difficult for athletes to improve their rank relative to their peers. Athletes might maintain their percent-rank but drop in overall ranking even if they complete repeated workouts at a faster rate due to similar improvements made by the average competitor. In this regard, maintaining one’s percent-rank across iterations of a workout should be viewed as a positive outcome, whereas improving upon one’s rank would mean the athlete enhanced their performance to an even greater degree than the remainder of the field. Athletes and coaches are advised to maintain perspective when identifying areas of need, and to focus on finding suitable pacing strategies that balance efficiency with physiological attributes when attempting to improve performance in specific workouts.
